# A Border Versus Non-Border Comparison of Food Environment, Poverty, and Ethnic Composition in Texas Urban Settings

**DOI:** 10.3389/fpubh.2015.00063

**Published:** 2015-04-28

**Authors:** Jennifer J. Salinas, Ken Sexton

**Affiliations:** ^1^School of Public Health, The University of Texas Health Science Center, Houston, TX, USA

**Keywords:** food environment, poverty, Hispanics, Texas, urban population

## Abstract

**Purpose:**

The goal was to examine the relationship between the food environment and selected socioeconomic variables and ethnic/racial makeup in the eight largest urban settings in Texas so as to gain a better understanding of the relationships among Hispanic composition, poverty, and urban foodscapes, comparing border to non-border urban environments.

**Methods:**

Census-tract level data on (a) socioeconomic factors, like percentage below the poverty line and number of households on foodstamps, and (b) ethnic variables, like percent of Mexican origin and percent foreign born, were obtained from the U.S. Census. Data at the census-tract level on the total number of healthy (e.g., supermarkets) and less-healthy (e.g., fast food outlets) food retailers were acquired from the CDC’s modified retail food environment index (mRFEI). Variation among urban settings in terms of the relationship between mRFEI scores and socioeconomic and ethnic context was tested using a mixed-effect model, and linear regression was used to identify significant factors for each urban location. A jackknife variance estimate was used to account for clustering and autocorrelation of adjacent census tracts.

**Results:**

Average census-tract mRFEI scores exhibited comparatively small variation across Texas urban settings, while socioeconomic and ethnic factors varied significantly. The only covariates significantly associated with mRFEI score were percent foreign born and percent Mexican origin. Compared to the highest-population county (Harris, which incorporates most of Houston), the only counties that had significantly different mRFEI scores were Bexar, which is analogous to San Antonio (2.12 lower), El Paso (2.79 higher), and Neuces, which encompasses Corpus Christi (2.90 less). Significant interaction effects between mRFEI and percent foreign born (El Paso, Tarrant – Fort Worth, Travis – Austin), percent Mexican origin (Hidalgo – McAllen, El Paso, Tarrant, Travis), and percent living below the poverty line (El Paso) were observed for some urban settings. Percent foreign born and percent Mexican origin tended to be positively associated with mRFEI in some locations (Hidalgo, El Paso) and negatively associated in others (Tarrant, Travis).

**Discussion:**

Findings are consistent with other studies that suggest the effects of Hispanic concentration on the foodscape may be positive (beneficially healthy) in border urban settings and negative in non-border. The evidence implies that the effects of Hispanic ethnic composition on the food environment are location-dependent, reflecting the unique attributes (e.g., culture, infrastructure, social networks) of specific urban settings.

## Introduction

Disparities in access to healthy food and related adverse health outcomes, such as obesity, diabetes, and cardiovascular disease, are the subject of ongoing research and public policy debates in the United States (1–7). There is a growing body of evidence indicating that the local food environment – availability of and access to healthy food choices at nearby community retail stores and restaurants – is a potentially important determinant of obesity and related health problems (8–16). Research has demonstrated that the local foodscape is an independent predictor of individuals’ food choices and diet quality, and that higher-income areas are associated with better access to supermarkets and a wider variety of healthful foods (9, 14, 17). Lower-income, primarily minority communities, on the other hand, are more likely to have little or no access to supermarkets, which obliges them to rely mainly on convenience stores and fast food restaurants that sell a more restricted range of healthy food items (1, 3, 10, 14, 15, 18, 19).

This reality has spawned research aimed at identifying and analyzing impacts from “food deserts” in impoverished, racially, and ethnically minority communities (1, 3, 4, 19). While there are various definitions of “food deserts’ depending upon the source, the United States Department of Agriculture (USDA) defines the term as a geographic area wherein access to affordable, quality, and nutritious foods is limited” (10). Additionally, in food-desert communities, the primary source of food may be fast food restaurants or convenience stores, and with few to no supermarkets within a reasonable driving distance; poor residents are left with only limited options to purchase fresh fruits or vegetables in these areas. As a result, it is often difficult for poor people living in a food desert to maintain a healthy diet because (a) they do not live in close proximity (one-mile for urban settings – 10 miles in rural areas) to supermarkets that sell healthy food and/or (b) they lack the financial resources to buy more-expensive healthy foods even if available (1, 3, 4, 10–12, 15). One area of particular concern is the southern region of Texas along the U.S.–Mexico Border, where high prevalence of obesity and related health disorders has been documented in a population that is predominately poor, Hispanic, environmentally challenged, and lacking knowledge of and access to healthy food options (20–27).

The purpose of this article is to assess whether variations in socioeconomic and ethnic context are associated with the nature of the food environment in urban settings across the State of Texas. Food environment data from the Centers for Disease Control and Prevention’s (CDC’s) modified retail food environment index (mRFEI) (28) is linked to socioeconomic and ethnic census-tract attributes from the 2010 U.S. Census (29) to characterize variations among eight urban locations. A comparison is made by border/non-border location to assess the potential contextual effect of socioeconomic context on food environment. The findings inform public health policies that aim to take account of differences in socioeconomic context and ethnic makeup among diverse urban surroundings in order to reduce disparities in access to healthy food.

## Materials and Methods

### Study setting

The analysis focuses on the eight most populated counties (i.e., surrogates for urban settings) in Texas. Each county represents a significant portion of the metropolitan area of one of the eight largest cities in Texas: Houston (Harris County), Dallas (Dallas County), Fort (Worth Tarrant County), Austin (Travis County), San Antonio (Bexar County), McAllen (Hidalgo County County), Corpus Christi (Nueces County), and El Paso (El Paso County). Sociodemographic characteristics, including race and Mexican ethnic concentration are summarized in Table 1. These metropolitan-area counties represent approximately 51.4% of the state’s total population and have diverse sociodemographic characteristics and race/ethnic composition, which provides ample contrast for the analysis.

**Table 1:** **Average census-tract mRFEI score and demographic characteristics by county**.

	mRFEI	% Below poverty line	Average # households on food stamps (mean + SD)	% Foreign born (mean + SD)	% Mexican origin (mean + SD)	% Bachelor’s or higher (mean + SD)	Median age	Total population
Total population								
Bexar (San Antonio)	9.2 (4.7)	22.0 (12.7)	271.7 (172.4)***	13.7 (6.4)***	59.2 (23.3)***	18.8 (16.8)***	34.2 (5.6)	1, 714, 773
Dallas	9.9 (6.0)	18.4 (12.1)	162.9 (140.5)*	22.8 (13.2)*	34.4 (24.3)	27.0 (22.9)	33.9 (6.7)	2, 368, 139
El Paso	13.8 (6.9)***	29.4 (15.4)***	352.7 (223.2)***	27.5 (9.6)	78.1 (18.5)***	17.5 (14.2)***	34.3 (5.2)	800, 647
Harris (Houston)	9.3 (6.4)	19.8 (13.2)	189.1 (147.3)	24.9 (12.8)	34.5 (25.5)	26.2 (23.6)	33.8 (6.3)	4, 092, 459
Hidalgo (McAllen)	14.2 (11.3)***	34.6 (13.6)***	572.1 (263.0)***	28.6 (7.5)	85.3 (10.3)***	17.0 (12.8)**	30.7 (5.4)**	774, 769
Nueces (Corpus Christi)	8.9 (6.0)	25.4 (14.2)*	340.1 (209.9)***	8.3 (5.1)***	63.4 (20.6)***	12.9 (11.3)***	35.8 (5.9)	340, 223
Tarrant (Fort Worth)	10.2 (5.1)	15.5 (12.2)***	165.3 (131.5)	15.7 (10.8)***	24.5 (21.7)***	27.6 (18.0)	35.2 (6.2)*	1, 809, 034
Travis (Austin)	9.4 (5.8)	22.1 (16.1)	188.6 (186.0)	19.2 (13.0)***	29.2 (20.7)*	43.3 (22.9)***	32.4 (6.1)*	1, 024, 266

*p < 0.05, **p < 0.01, ***p < 0.001.

### The modified retail food environment 2008

The mRFEI (28) is a measure of the total number of healthy and less-healthy food retailers in a census tract. The distinction between healthy versus less-healthy (e.g., unhealthy) is based on available food offerings from retail establishments, such as grocery stores, convenience stores, and fast-food restaurants. The mRFEI identifies the percent of all the food retailers in a given census tract, that are considered to provide healthy food choices. The potential census-tract score for the mRFEI ranges from 0 or “food desert” to 100 or “ideally healthy” – although few across the country are at this level.

### U.S. census 2010

The census-tract data were obtained through the U.S. Census Bureau, specifically the American fact finder website (29). Demographic and social characteristic tables were selected and downloaded in a delimited format together with the annotation file. The tables were screened and variables of interest selected and merged into one file in excel format. A database was created with the census-tract FIPS as the ID indicator. Variables used for this analysis were total population, median age, percent below the poverty line, percent foreign born, percent of Mexican origin, and percent of families on food stamps.

### Analysis

Descriptive statistics were generated for average mRFEI Score, total population, median age, average percent below the poverty line, average percent adults 25+ with a bachelor’s degree or higher, average percent foreign born, average percent Mexican origin, and average percent of families on food stamps by each urban setting. A mixed effects (multilevel) model was used to test for significant variation between urban settings on the basis of the relationship between socioeconomic and ethnic context and mRFEI scores. We performed linear regression to identify which factors were significant for each urban setting. To account for significant clustering identified in the mixed-effect modeling, the jackknife variance estimate was used. The jackknife variance estimate accounts for clustering and autocorrelation of adjacent census tracts by running repeated models while randomly removing cases each iteration to improve the precision of the variance estimate that could be biased due to significant clustering within each urban setting. Interaction models were generated between urban settings and census-tract socioeconomic and ethnic variables.

## Results

Descriptive statistics for each urban setting are presented in Table 1. Harris County (Houston) was selected as the reference category due to the fact it is the largest urban setting examined. Results show relatively small variation in the food environment and substantial variation in socioeconomic and race/ethnic composition across major urban areas in the state of Texas. Overall, average mRFEI scores are relatively low in each of the eight urban settings, ranging from 8.9 in Nueces County (Corpus Christi) to 14.2 in Hidalgo County (McAllen). The range for all counties is from 1.8 to 66.7, which suggests that while there are some census tracts that are closer to the “ideal,” most are in the unhealthy range. Both El Paso and Hidalgo – each located on the Texas–Mexico border – had the highest average mRFEI scores: 13.8 (*p* < 0.001) and 14.2 (*p* < 0.001), respectively, and were the only counties with significantly different values from Harris County. El Paso (29.4%, *p* < 0.001), Hidalgo (34.6%, *p* < 0.001), and Nueces (25.4%, *p* < 0.05) had a significantly higher average census-tract percent below the poverty line compared to Harris County, whereas Tarrant County (Fort Worth) (15.5%, *p* < 0.05) had a significantly lower average. Hidalgo County (572.1, *p* < 0.001) and El Paso (352.7, *p* < 0.001) had the highest census-tract average households on food stamps, followed by Nueces County (340.1, *p* < 0.001), Bexar County (San Antonio) (271.7, *p* < 0.001), and Dallas County (162.9, *p* < 0.05). Travis County (Austin) had the highest average% Bachelor’s degree or higher (43.3%, *p* < 0.001). Bexar (18.8%, *p* < 0.001), El Paso (17.5%, *p* < 0.001), Hidalgo (17.0%, *p* < 0.01), and Nueces (12.9%, *p* < 0.001) Counties had significantly lower average census-tract percent with a Bachelor’s degree or higher compared to Harris County. While both Hidalgo (30.7, *p* < 0.01) and Travis Counties (32.4, *p* < 0.05) had significantly lower census-tract median age, Tarrant (35.2, *p* < 0.05) had significantly higher census-tract median age than Harris County.

In terms of race/ethic composition, there were no significant differences between El Paso, Hidalgo, and Harris Counties in the average census-tract percent foreign born. However, Bexar (13.7, *p* < 0.001), Dallas (22.8, *p* < 0.05), Nueces (8.3%, *p* < 0.001), Tarrant (15.7%, *p* < 0.001), and Travis (19.2%, *p* < 0.001) all had significantly lower average census-tract percent foreign born. This is contrasted with percent Mexican origin, where both El Paso (78.1%, *p* < 0.001) and Hidalgo (85.3%, *p* < 0.001) were predominantly Mexican origin. In addition, while in Bexar (59.2%, *p* < 0.001) and Nueces Counties (63.4%, *p* < 0.001) the majority population was of Mexican origin, both Tarrant (24.5%, *p* < 0.001) and Travis (29.2%, *p* < 0.05) had a significantly lower percent Mexican origin than Harris County.

Table 2 presents mixed effects and linear regression results for socioeconomic and ethnic characteristics by urban setting. In the mixed effects model, % foreign born was associated with a lower mRFEI score (β = −6.94, *p* = 0.000), whereas % Mexican origin was associated with a higher mRFEI (β = 5.71, *p* = 0.000). No socioeconomic variables were significantly associated with mRFEI in the mixed-effects model. The linear regression using jackknife variance estimate yields similar significant coefficients for % foreign born (β = −7.5, *p* = 0.000) and % Mexican origin (β = 5.58, *p* = 0.000), with no other significant results. In terms of comparing our seven urban settings (i.e., counties) to Harris County (Houston), the census-tract average mRFEI in Bexar was 2.12 less than Harris (*p* = 0.001), while the value in El Paso was 2.79 (*p* = 0.004) higher than Harris. The only other statistically significant difference (*p* = 0.036) from Harris was Neuces County, which was 2.90 less.

**Table 2:** **Regression results for mRFEI**.

	Mixed effects (clustered by county)	Linear regression w/Jackknife variance test
**Sociodemographics**
% Below poverty line	−0.029 (0.102)	−0.029 (0.189)
Average # households on food stamps	−0.001 (0.324)	−0.002 (0.390)
% Foreign born	−6.94 (0.000)	−7.5 (0.000)
% Mexican origin	5.71 (0.000)	5.58 (0.000)
% Bachelor’s or higher	−0.002 (0.863)	−0.002 (0.884)
Median age	−0.019 (0.571)	−0.023 (0.536)
**County (Ref. Harris i.e., Houston)**		
Bexar (San Antonio)		−2.12 (0.001)
Dallas		0.376 (0.403)
El Paso		2.79 (0.004)
Hidalgo (McAllen)		3.28 (0.062)
Nueces (Corpus Christi)		−2.90 (0.036)
Tarrant (Fort Worth)		0.599 (0.240)
Travis (Austin)		−0.019 (0.977)

**Figure 1:**
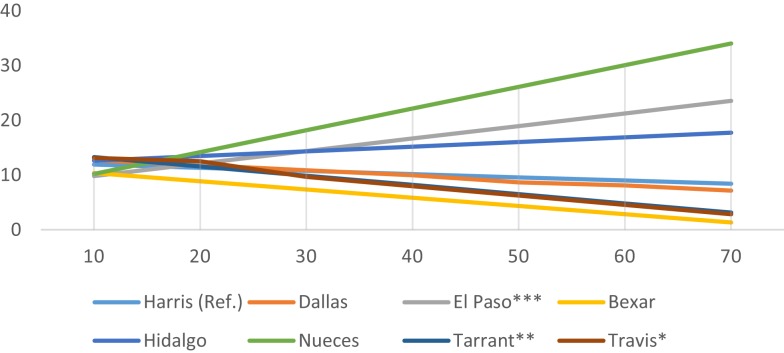
**Interaction effects between county and percent foreign born**.

**Figure 2:**
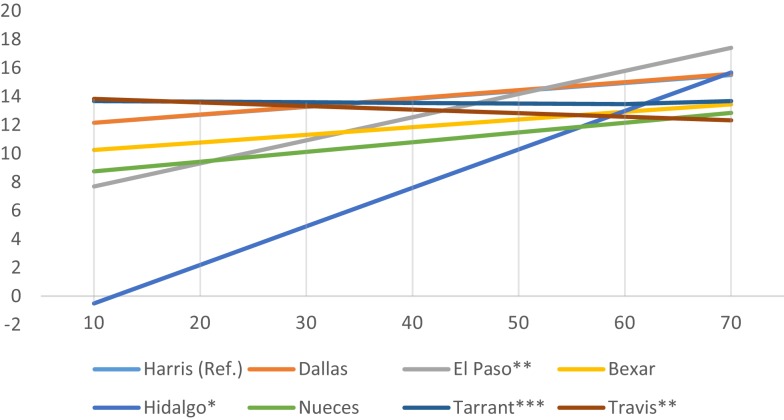
**Interaction effects between county and percent Mexican origin**.

**Figure 3:**
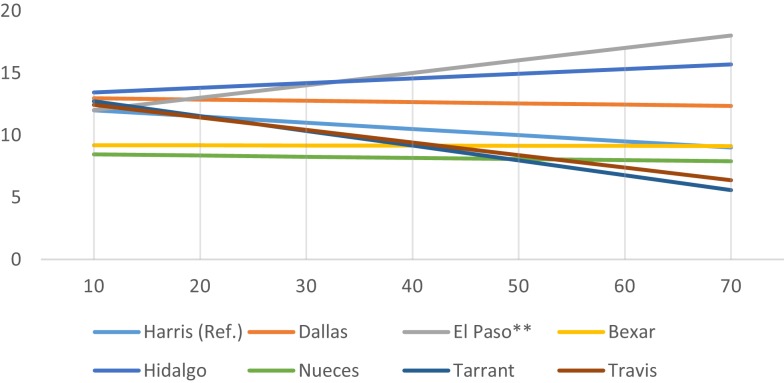
**Interaction effects between county and percent living below the poverty line**.

While county average census-tract mRFEI scores are uniformly low (range 8.9–14.2), significant county-level interaction effects were observed. Figures 1–3 present only the significant interaction results between urban setting and (a) % foreign born (Figure 1), (b) % Mexican origin (Figure 2), and (c) % living below the poverty line (Figure 3). As shown in Figure 1, there is a diverging trend in the association between urban setting and % foreign born; whereas in the border (El Paso, Hidalgo) or near- border counties (Bexar, Nueces), there is a positive association between % foreign born and the mRFEI. Conversely, there is a negative association for the non-border counties (Harris, Dallas, Tarrant, Travis). Statistically significant interaction effects were observed only for El Paso (*p* < 0.001), Tarrant (*p* < 0.01), and Travis (*p* < 0.05). In Figure 2, % Mexican origin was only significant for Hidalgo, El Paso, Tarrant, and Travis. However, similar to what was observed in the % foreign born interaction model, % Mexican origin was positively associated with mRFEI in Hidalgo and El Paso, and negatively associated in Tarrant and Travis, although the pattern is not as salient. Percent poverty (Figure 3) is the only significant socioeconomic variable interaction with urban setting, and only for El Paso, where % poverty is positively associated with the mRFEI score.

## Discussion

The purpose of this study was to elucidate the relationship between socioeconomic and ethnic context and food environment in the largest urban settings within Texas. Findings revealed that the relationship between socioeconomic characteristics, ethnic context and food environment varies by urban setting, and depends on border/non-border location. Access to healthy food options is said to differentially place certain race/ethnic groups and socioeconomic strata at higher-than-average risk for obesity and related adverse health outcomes (1, 3, 10, 15, 18, 19, 24–26). This is exemplified in the Texas–Mexico border region, where there is a high concentration of poverty and persons of Mexican origin, coupled with a higher–than-average prevalence of obesity (30), which places Mexican American residents in the region at increased risk for diabetes and uncontrolled hypertension (20–27). The findings from this study suggest that the impact of race/ethnic concentration and socioeconomics on food environment may be a function of context and geographic location, rather than socioeconomics and ethnic composition alone.

While few studies have compared border to non-border food environments, the findings from this study are consistent with what is known, and contribute to mounting evidence that contextual influences may differ based on geographic proximity to the border and therefore be responsible for risk variation in obesity and related chronic diseases. For example, Salinas et al. (26) using the same data by census tract across the State of Texas found that census tracts in the U.S.-Mexico border region have, on average, better food environments than non-border census tracts. Nonetheless, the relationship between the mRFEI and % foreign born and % families on food stamps varied by border/non-border location (26). In this study, the border and near-border urban settings had higher mRFEI scores and higher Hispanic ethnic and immigrant concentration was associated with better food environments. In non-border urban settings, these same factors were associated with lower mRFEI scores.

Hispanic concentration on the Texas–Mexico border is known to be among the highest in the United States (29); at the same time, obesity rates in some areas of the region are well-above national averages (29). These statistics suggest that food environment might also be less healthy in urban settings on the Texas–Mexico border than in non-border urban settings further north. Yet, individual-level studies have found, instead, a protective effect for Hispanic ethnic concentration and health (31–33). For example, evidence from the Multi-Ethnic Study of Atherosclerois (MESA) study suggests that higher ethnic concentration may be protective from obesity, and that movement to ethnically mixed communities may be a significant risk factor for weight gain in ethnic minority groups (34). Additionally, Eschbach et al (35) using SEER data found a protective effect of Hispanic ethnic concentration and cancer, a condition known to be associated with obesity. The findings from this study provide evidence for a potential mechanism underlying the salubrious effect of very high Hispanic concentration, which may be related to contextual factors that influence food environment. It is plausible that in Hispanic-majority communities Hispanics have access to more and better resources that promote better health, compared to similar communities where they are the minority. This higher concentration may translate into greater access to fruits and vegetables; however, access alone may not reduce the risk of obesity in border urban settings relative to non-border urban settings in Texas, suggesting additional contextual factors at play, such as social context, group dynamics, and cultural traditions that become important in communities where Hispanics are in the majority.

One of the more common explanations for the health benefits of Hispanic communities or high ethnic concentration has been the potential benefit of immigrants who bring their “more healthy” beliefs or customs to their destinations. The traditions brought from their countries of origin provide resources to promote better health such as stores and restaurants that may serve or sell more traditional food. The evidence on immigrant concentration has been mixed. Some studies suggest that higher immigrant concentration can be, at the same time, both a risk factor and a protective attribute for disease and mortality (36, 37). For example, Omariba and colleagues (36) found an association between higher immigrant concentration and hospital admissions for lower cardiovascular disease in Ontario, Canada. However, while immigrant concentration in El Paso has been found to be protective from asthma symptoms (37), in Los Angeles Hispanic infants born to mothers who lived in higher immigrant concentration communities were more like to die than those whose mothers lived in non-immigrant enclaves (4). In the study presented here, the positive and marginal benefit of immigrant concentration on food environment occurred in border or near-border urban settings (El Paso and Hidalgo), while in non-border urban settings immigrant concentration was associated with a less healthful food environment. Although our study made use of aggregated data, the results are consistent with previous studies, and add to the mounting evidence suggesting that the health benefit or harm derived from an immigrant enclave may depend on the location-specific context of each community, including variations in the food environment.

Poverty is often cited as one of the largest risk factors for unhealthy urban food environments (38, 39). Evidence suggests that persons who live in poor communities need to travel further to obtain healthy or fresh food (40), and there is a strong association between fruit and vegetable availability and community socioeconomic environment (18). Most of the existing studies have examined a single urban area or focused on a specific racial or ethnic group without taking into consideration the overall ethnic environment relative to other settings. In this study, poverty was the only socioeconomic condition significantly associated with the mRFEI; but only in El Paso. Percent living below the poverty line in El Paso was positively associated with the mRFEI, which is contrary to conventional expectations that poorer communities would tend to have fewer healthy food options. While El Paso is a relatively large metropolitan area, it is still less developed that the referent category, Harris County (Houston). It may be that access to less-healthy food in poorer communities depends on geospatial factors, like the level and nature of development and infrastructure in place within a given urban setting. This is an area of research that has been under explored and more information is needed.

Although this study provides additional evidence supporting the salubrious effects of Hispanic ethnic composition, at the same it provides inconsistent results in relation to what is already known about the link between socioeconomics and food environment. It is important to keep in mind that this study has certain limitations that should be taken into account when interpreting its main findings. First, since these data were aggregated at the population level we are unable to make inferences about individual beliefs or behaviors. So while the food environment may vary between urban settings, we still do not know the impact it has on actual access or consumption. Understanding the relationship between ethnic concentration, socioeconomic context and food environment is an essential next step necessary to establish the causal chain between context, individual behavior, and obesity and chronic disease risk.

An additional limitation to take into consideration is that the mRFEI measures the proportion of food vendors that sell healthy food relative to unhealthy food. While this measure provides a picture of the geographic distribution of food availability, it does not distinguish what type of foods are actually sold at a given location or their cost. Moreover, the mRFEI provides a score for each individual census tract and does not take into consideration adjacent census tracts or travel distances to access supermarkets or healthier food vendors. Although this analysis only involved urban settings (thereby eliminating distance variation between urban versus rural differences), local ordinances and building restrictions could influence the concentration of supermarkets relative to fast food restaurants or convenience stores. Therefore, variations in local zoning in the border urban settings versus non-border settings may have influenced the findings from this study, an important factor to consider in future work.

Despite these limitations, this study does provide useful information germane to future directions in both relevant research and effective policy. Understanding how the food environment influences actual behaviors of food consumption is an important direction to take in future research. Hispanic populations are overrepresented among those who are overweight or obese. They are also overrepresented among those living in poverty. The food environment has a direct relationship with both poverty and food access, and has implications for subsequent overweight or obesity outcomes. Still, the processes that link context to obesity risk are not well understood. In order to address the issues of obesity in Hispanic communities, particularly those on the border, it is important to acknowledge the significance of the food environment and find ways to increase access to healthy foods while at the same time limiting access to unhealthy foods. Ultimately, fighting obesity depends on making lifestyles changes, which are likely to be more successful in food environments that support healthy living.

## Conflict of Interest Statement

The authors declare that the research was conducted in the absence of any commercial or financial relationships that could be construed as a potential conflict of interest.
